# Cowpea Mosaic Virus Immunotherapy Combined with Cyclophosphamide Reduces Breast Cancer Tumor Burden and Inhibits Lung Metastasis

**DOI:** 10.1002/advs.201802281

**Published:** 2019-06-19

**Authors:** Hui Cai, Chao Wang, Sourabh Shukla, Nicole F. Steinmetz

**Affiliations:** ^1^ Department of NanoEngineering/Department of Radiology/Moores Cancer Center/Department of Bioengineering University of California, San Diego La Jolla CA 92093 USA; ^2^ Department of Biomedical Engineering Case Western Reserve University 10900 Euclid Avenue Cleveland OH 44106 USA

**Keywords:** cancer immunotherapy, chemotherapy, in situ vaccination, triple‐negative breast cancer

## Abstract

Patients with metastatic triple‐negative breast cancer (TNBC) have a poor prognosis, so new therapies or drug combinations that achieve more effective and durable responses are urgently needed. Here, a combination therapy using cowpea mosaic virus (CPMV) and low doses of cyclophosphamide (CPA) is developed with remarkable synergistic efficacy against 4T1 mouse tumors in vivo. The combination therapy not only attenuates the growth of primary tumor and increases survival, but also suppresses distant tumor growth and reduces lung metastasis. Mechanistic analysis indicates that the combination of CPMV and CPA increases the secretion of several cytokines, activates antigen‐presenting cells, increases the abundance of tumor infiltrating T cells, and systematically reverses the immunosuppression. These results show that the combination of CPMV in situ vaccination with chemotherapy may become a potent new strategy for the treatment of TNBC.

## 1 Introduction

Breast cancer is the most common form of cancer in women and is second only to lung cancer in terms of cancer‐related mortality.^1^ Triple‐negative breast cancer (TNBC) represents 15–20% of all breast cancer cases, with a worse prognosis and a higher recurrence rate (≈11%) than other subtypes (≈6%) of the disease.^2^, ^3^ No targeted therapies for TNBC are currently available, and current treatments often fail to slow tumor progression, with fatal consequences.^4^, ^5^ Conventional chemotherapy remains the primary treatment option,^6^, ^7^, ^8^ and neoadjuvant chemotherapy has proven efficacious, but only in a portion of chemo‐sensitive TNBC patients.^9^, ^10^ Despite comprehensive and aggressive management, more than 50% of patients suffer recurrence and more than 37% succumb to cancer within five years.^6^ New therapeutic strategies and drug combinations for the effective treatment of TNBC are therefore urgently required.

Cancer immunotherapy remodels the host immune system to eradicate tumor cells, and has become a promising approach to address TNBC.^11^ For example, the immune checkpoint inhibitors pembrolizumab (Pembro) is clinically efficacious in metastatic TNBC patients with PD‐L1^+^ tumors and elevates high levels of tumor‐infiltrating leukocytes.^12^, ^13^ Although these clinical responses are durable, the overall response rates remain low (19–23%).^14^ More importantly, adverse events from immune checkpoint inhibitors are a common occurrence, including the cardiotoxic effects that often have serious complications with a relatively high mortality.^15^ Therefore, safe but effective therapeutic strategies are needed to significantly strengthen those responses that do occur, covert nonresponders to responders, and overcome acquired resistance to immunotherapy. Viruses including plant viruses and phages have been used as carriers for targeted cancer drug delivery.^16^, ^17^ A newer direction is their application as immunomodulatory agents and application in cancer immunotherapy.^18^ Recently, we have shown that cowpea mosaic virus (CPMV) can induce antitumor responses in several murine models of cancers when introduced into a tumor microenvironment (TME) as an in situ vaccine.^19^, ^20^, ^21^ The virus activates the innate immune response, recalibrating the cancer–immunity cycle to eliminate cancer cells via the adaptive immune system.

Here, we investigated CPMV in situ vaccination combined with cyclophosphamide (CPA) chemotherapy to treat triple‐negative 4T1 murine breast tumors. We chose CPA because it is the most widely used alkylating agent for chemotherapy and has extensive immunomodulatory activity,^22^ although high doses cause immunosuppression.^23^ The immunomodulatory effects may involve a Th2/Th1 shift in cytokine production, depletion of tumor‐induced suppressor T‐cell populations, long‐term survival and proliferation of lymphocytes, induction of soluble mediators, and/or the resetting of dendritic cell (DC) homeostasis.^23^, ^24^ Moreover, low doses of CPA induce pro‐immunogenic activity in tumor cells, including the hallmarks of immunogenic cell death (ICD).^25^

We hypothesized that CPMV combined with CPA would act synergistically to induce immunomodulatory effects that would debulk the 4T1 tumor to provide a burst of tumor antigens in the context of ICD, thus promoting the recognition and processing of those antigens. The stimulation of the immune system by CPMV would augment this antitumor immunity via efficient processing and presentation of tumor antigens and induce memory to prevent metastasis and recurrence. We therefore evaluated the efficacy of CPMV and CPA against 4T1 murine tumors and metastases, and assessed the antitumor immune response triggered by this combination therapy.

## 2 Results

### 2.1 CPMV and CPA Monotherapy Moderately Inhibit 4T1 Tumor Growth

**Figure 1:**
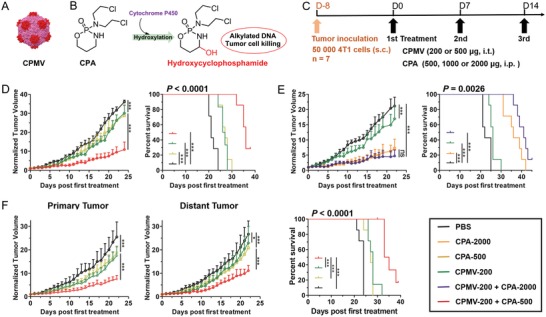
Combination therapy using CPMV and CPA reduces the tumor burden and improves survival in 4T1 tumor‐bearing mice. A) CPMV. B) Conversion of CPA to its activated form. C) Treatment schedule. D) Combination therapy (CPMV with low‐dose CPA) shows synergistic efficacy. E) Combination therapy (CPMV with high‐dose CPA) shows little synergistic efficacy. F) Combination therapy (CPMV with low‐dose CPA) shows synergistic effects against a bilateral 4T1 tumor model. Tumor growth curves show average of normalized tumor volume and standard deviation, with statistical analysis by two‐way ANOVA (*p < 0.05, ***p < 0.001, ns = not significant). Statistical analysis for survival curves: log‐rank (Mantel–Cox) test (***p < 0.001).

Mammary carcinoma 4T1 is derived from BALB/c mice and shares many characteristics with naturally occurring human TNBC.^26^ It is therefore widely used as a syngeneic tumor model for the assessment of novel therapeutic approaches.^27^ We evaluated the activity of single‐agent CPMV against 4T1 tumors (**Figure** **1**A). The 4T1 cells were subcutaneously (s.c.) inoculated into the right flank of BALB/c mice. When the tumor size reached 30–50 mm^3^, CPMV was delivered intratumorally (i.t.) at doses of 200 and 500 µg per injection. The treatments were administered three times at intervals of 7 days (Figure 1C). We found that the dose of 200 µg CPMV was sufficient, and that the higher dose did not improve the efficacy based on tumor growth curves (Figure S1A,B, Supporting Information). However, CPMV alone had a limited efficacy against 4T1 tumors regardless of the dose, which is not surprising given that most solo immunotherapies do not eradicate 4T1 tumors and combination therapy is required to achieve this outcome.^12^, ^13^, ^28^

We also evaluated the monotherapeutic efficacy of CPA, which must be activated by hepatic cytochrome P450 to generate its toxic form, 4‐hydroxycyclophosphamide (Figure 1B). CPA was delivered intraperitoneally (i.p.) at doses of 500, 1000, and 2000 µg per injection (corresponding to 25, 50, and 100 mg kg^−1^, respectively) following the same schedule (Figure 1C). The efficacy of CPA was dependent on the dose (Figure S1B,C, Supporting Information). Tumor growth was modestly inhibited by CPA at low doses (500 and 1000 µg) but significantly inhibited at the higher dose of 2000 µg. However, high doses of CPA can significantly increase the risk of side effects and immunosuppression.^29^ Many studies have shown that antitumor immunity plays a key role in the control of tumor growth after chemotherapy,^25^ so chemotherapy‐mediated immunosuppression should be avoided when immunotherapy is the goal. These findings together with our preliminary results from monotherapy treatments encouraged us to study new therapeutic strategies involving the combination of CPMV and CPA.

### 2.2 Combination Therapy Is More Efficacious and Induces a Systemic Antitumor Response

To investigate the efficacy of combination therapy, 4T1 tumor‐bearing mice were randomized to one of four treatment groups: i) PBS, ii) CPMV (200 µg, i.t.), iii) CPA (500 µg, i.p.), and iv) CPMV (200 µg, i.t.) + CPA (500 µg, i.p.), delivered at the same time. As described above, single‐agent treatments inhibited tumor growth only slightly (Figure 1D). However, combination therapy significantly improved the efficacy, resulting in the substantial inhibition of tumor growth (Figure 1D; Figure S2, Supporting Information). On day 24, the normalized tumor volume in the PBS group was 3‐fold larger than the combination therapy group, but only 1.2‐fold larger compared to each of the monotherapy groups. The combination therapy also achieved a significant improvement in survival (Figure 1D), with median survival extended to 36 days, significantly better than the 21 days in the PBS group, and the 26 and 27 days in the CPMV and CPA monotherapy groups, respectively. These results suggest that low‐dose CPA synergizes with CPMV to enhance the antitumor immune response. We increased the dose of CPA to 2 mg and again evaluated the efficacy of combination therapy. Compared to high‐dose CPA monotherapy, the combination therapy with high‐dose CPA did not significantly improve the efficacy (Figure 1E; Figure S3, Supporting Information), suggesting that the immunomodulatory and pro‐immunogenic effects induced by low doses of CPA are necessary for the synergistic activity. Thus, we focused on the low‐dose CPA and its combination with CPMV for further treatments and mechanistic studies.

We next used a bilateral 4T1 model to evaluate the efficacy of combination therapy against a distant secondary tumor that was inoculated at the same time as the primary tumor, but not directly treated by CPMV in situ vaccination. CPMV or low‐dose CPA monotherapy had little impact on the distant tumor (Figure 1F). Interestingly, the injection of CPMV directly into the primary tumor in combination with CPA showed synergistic efficacy against the distant tumor, resulting in the significant inhibition of distant tumor growth and extended survival in the bilateral model (Figure 1F; Figures S4 and S5, Supporting Information). These results indicated that systemic antitumor immune responses were induced by the combination of low‐dose CPA and CPMV‐mediated in situ vaccination of the primary tumor, leading to the suppression of the distant tumor.

### 2.3 CPMV Combined with Mafosfamide Shows No Synergistic Efficacy against a Distant Tumor

**Figure 2:**
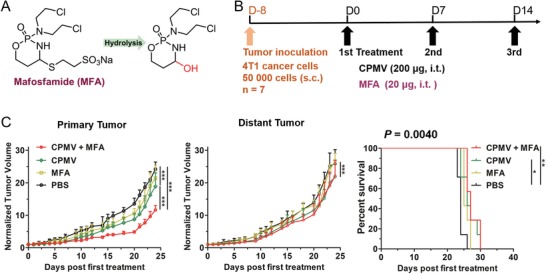
Combination therapy using CPMV and MFA shows no synergistic efficacy against the bilateral 4T1 model. A) Conversion of MFA to hydroxyclophosphamide by hydrolysis. B) Treatment schedule. C) Tumor growth and survival curves. Tumor growth curves show average of normalized tumor volume and standard deviation, with statistical analysis by two‐way ANOVA (***p < 0.001). Statistical analysis for survival curves: log‐rank (Mantel–Cox) test (ns = not significant, *p < 0.05, **p < 0.01).

In the above treatment of bilateral 4T1 tumors, CPA was administered i.p. and thus has an equivalent impact on the primary and distant tumors, such as the induction of ICD to increase immunogenicity together with immunomodulatory effects.^23^, ^24^, ^25^ To understand whether systemic exposure to CPA is necessary for its synergistic efficacy against the distant tumor, we combined CPMV with mafosfamide (MFA), the 4‐thioethane sulfonic acid salt of 4‐hydroxy‐cyclophosphamide, which is a preactivated CPA analog.^30^ Unlike CPA, MFA does not require hepatic activation and can spontaneously hydrolyze to form 4‐hydroxy‐cyclophosphamide (**Figure** **2**A). MFA can therefore be delivered directly into the tumor together with CPMV by intratumoral injection, avoiding systemic exposure and minimizing the impact of chemotherapy on the distant tumor. Following a similar schedule (Figure 2B), we investigated the effect of this regimen in the bilateral 4T1 model, combining CPMV (200 µg, i.t.) and very low overall dose of MFA (20 µg, i.t.). As seen with CPA and CPMV, the combined therapy was moderately more efficacious than either monotherapy against the primary tumor (Figure 2C; Figure S6, Supporting Information), while no synergistic efficacy was observed against the distant tumor. Thus, the treatment group survived only marginally longer than the PBS group (Figure 2C). These results indicate that systemic exposure to chemotherapy is necessary to induce synergistic efficacy against the distant tumor, implying the antitumor immune response is dependent on the immunogenic impact of systemic chemotherapy.

### 2.4 A Combination of CPMV and CPA Reduces 4T1 Metastasis

**Figure 3:**
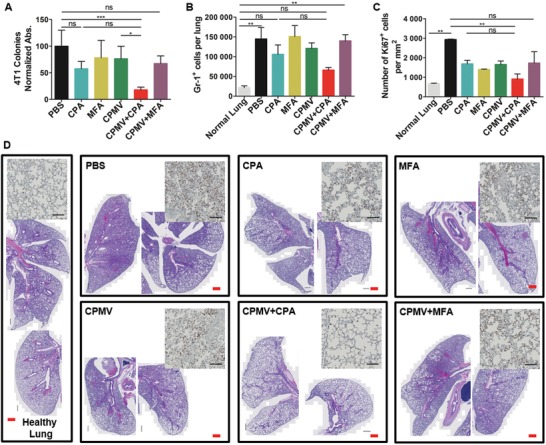
Combination therapy using CPMV and CPA reduces lung metastasis. A) Normalized absorbance of crystal violet in different treatment groups (*n* = 4). (Lungs were harvested 8 days after the first treatment.) B) The number of Gr‐1^+^ granulocytes in the lung (*n* = 4, harvested 8 days after the first treatment) determined by flow cytometry. C) Abundance of dividing cells (Ki67 positive) in the lung sections (*n* = 2). D) Representative lung sections stained with H&E (Scale bar (red) = 500 µm). The inserted images are the representative immunochemical stain of Ki67 antibody (Scale bar = 100 µm). The data represent means and standard deviations, with statistical analysis by one‐way ANOVA (ns = not significant, **p < 0.01).

Metastatic disease develops spontaneously in the lungs of mice with a 4T1 primary tumor as early as 8 days after inoculation, mimicking the metastases observed in human TNBC.^31^ We therefore investigated whether the combination of CPMV and CPA would prevent 4T1 lung metastasis. Following the treatments described above for the bilateral model, lung cells were harvested from tumor‐bearing mice 8 days after the first treatment and were cultured in the presence of 60 × 10^−6^ m 6‐thioguanine for 10 days. Colonies indicative of 4T1 cancer cells were revealed by staining with 0.1% w/v crystal violet. The quantitative results show that there was little difference in 4T1 colonies between the PBS control, the MFA and CPMV monotherapy groups, and the CPMV+MFA combination therapy group (**Figure** **3**A). However, CPA monotherapy reduced the number of 4T1 colonies to 60% compared to the PBS group, and the CPMV+CPA combination therapy reduced the number of 4T1 colonies to 20% compared to the PBS group, indicating the greater efficacy of this combined treatment for the prevention of lung metastasis.

Lung metastasis in 4T1 tumor‐bearing mice is characterized by the infiltration of myeloid‐derived suppressor cells into the lung tissue.^32^ We therefore determined the abundance of Gr‐1^+^ myeloid cells in lung samples by flow cytometry. Compared to the lungs from healthy mice, there was a significant increase (sixfold) in the number of Gr‐1^+^ myeloid cells present in lungs from 4T1 tumor‐bearing mice treated with PBS (Figure 3B). A similar increase was observed in the MFA and CPMV monotherapy groups, and in the mice treated with the combination of CPMV and MFA. However, in 4T1 tumor bearing mice treated with CPA or the combination of CPMV and CPA, the number of Gr‐1^+^ myeloid cells was reduced to half of that in PBS treated mice (Figure 3B). We next performed histological analysis of lung tissue harvested on day 8 post first treatment. Due to the lack of specific marker for metastatic 4T1 cancer cells, we quantified the cancer cells in the lung tissue by immunohistochemical analysis of Ki67 antibody that stains for proliferation nuclei. Quantification of stained sections showed a significant reduction in Ki67^+^ cells in lungs from CPMV+CPA combination therapy as compared to the PBS treated group (Figure 3C). This is in agreement with the trends observed for 4T1 colonies in lung tissues (Figure 3A) and confirms that CPMV+CPA combination therapy is more efficient reducing 4T1 lung metastasis. Furthermore, hematoxylin and eosin (H&E) staining (Figure 3D) also revealed the presence of significantly fewer infiltrating myeloid cells in the CPMV+CPA combination therapy group, as indicated by the larger alveolar spaces and less widespread purple stain compared to other groups. Together, these results indicate that CPMV+CPA combination therapy inhibits 4T1 metastasis with greater efficacy than either monotherapy.

### 2.5 Cytokine Profiling

**Figure 4:**
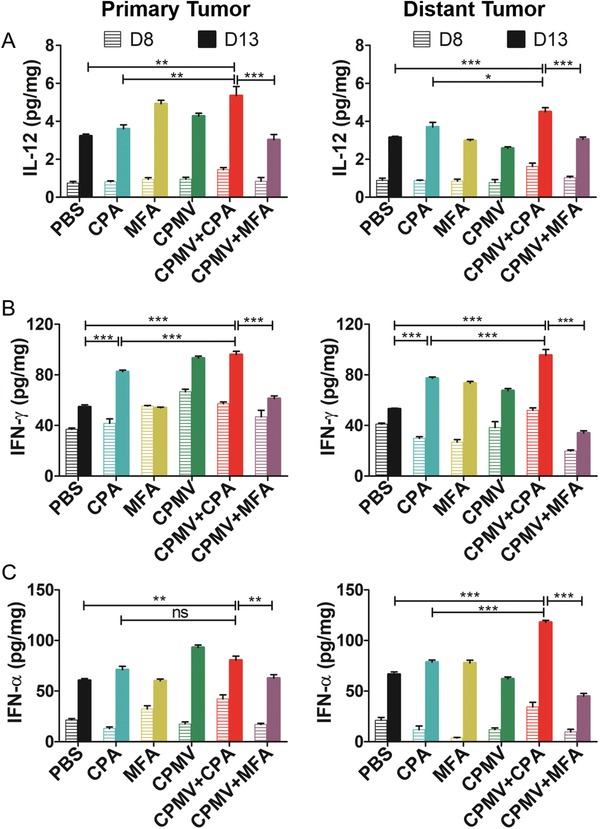
Cytokine profiles in tumor tissue harvested 8 and 13 days after the first treatment indicated that the CPMV+CPA combination therapy upregulated the cytokines A) IL‐12, B) IFN‐γ, and C) INF‐α in both the primary and distant tumors. Cytokine levels were determined by ELISA, with statistical analysis by one‐way ANOVA with Tukey's test (**p < 0.01, ***p < 0.001).

Cytokines are secreted by immune cells and can influence the behavior of cells around them. Therefore, cytokine profiling in the TME may provide important clues about the immunomodulatory effect induced by the treatment regimens described above. We measured cytokine levels ex vivo by enzyme‐linked immunosorbent assay (ELISA) in homogenized tumor lysates collected 8 and 13 days after the first treatment. We focused on the cytokines that positively influence the antitumor response: IL‐12, IFN‐α, and IFN‐γ. The normalized expression levels of the cytokines are shown in **Figure** **4**. The general trend was that the secretion of these cytokines increased from day 8 to 13. The expression of IL‐12 increased significantly in both the primary and distant tumors in response to the CPMV+CPA combination therapy (Figure 4A), whereas only the primary tumor showed higher IL‐12 levels in response to monotherapy. The upregulation of IL‐12 by CPMV probably reflected the uptake of the virus particles by antigen‐presenting cells (APCs), whereas the upregulation of IL‐12 by CPA or MFA reflected the immunomodulatory effect of chemotherapy. IL‐12 is mainly secreted by APCs and can bridge the innate and adaptive immune responses.^33^ Therefore, the increased expression of IL‐12 indicated that the APCs were activated and potentiated to induce the adaptive immune response. IL‐12 is also the major driver of Th1 differentiation and potently stimulates the production of IFN‐γ to coordinate natural mechanisms of antitumor defense.^34^ We found that monotherapy with CPMV or CPA increased the expression of IFN‐γ in both the primary and distant tumors, whereas treatment with MFA did not have this effect (Figure 4B). However, the CPMV+CPA combination therapy significantly increased the expression of IFN‐γ in both the primary and distant tumors, which was not achieved by the CPMV+MFA combination (Figure 4B). We also investigated the release of IFN‐α, a type I interferon that can activate immune cells and support the antitumor response. The expression of IFN‐α increased in the primary tumor following treatment with CPMV or CPMV+CPA, and in the distant tumor following the CPMV+CPA combination therapy (Figure 4C). It is interesting that CPMV was able to synergize with CPA but not MFA to induce these cytokines in both primary and distant tumors. Indeed, we found that MFA actually counteracted the induction of cytokines by CPMV in the context of the CPMV+MFA combination therapy, probably due to the different dose of MFA compared to CPA and differences in drug metabolism kinetics. The cytokine profiling data indicated that CPMV+CPA combination therapy triggered a more profound increase in the expression of these cytokines in the TME, particularly in the distant tumor. In summary, the greater expression of IL‐12, IFN‐γ, and IFN‐α indicated that APCs were activated and potentiated to induce an adaptive immune response.

### 2.6 Cellular Tumor Infiltration

**Figure 5:**
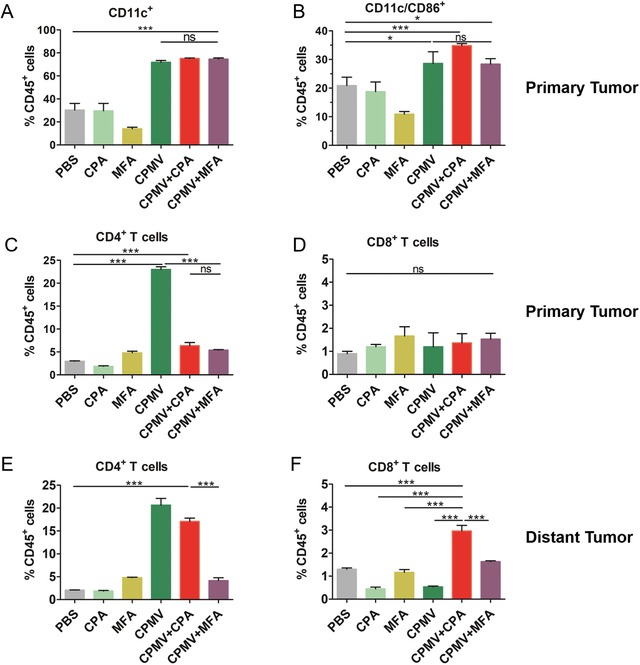
Abundance of different populations of immune cells in the tumor microenvironment. A) Tumor‐infiltrating dendritic cells (CD11c^+^) in the primary tumor on day 8. B) Tumor‐infiltrating activated dendritic cells (CD11c/CD86^+^). C,D) Tumor‐infiltrating CD4^+^ and CD8^+^ T cells in the primary tumor on day 13. E,F) Tumor‐infiltrating CD4^+^ and CD8^+^ T cells in the distant tumor on day 13. Statistical analysis by one‐way ANOVA with Tukey's test (**p < 0.01, ***p < 0.001).

To better understand the synergistic mechanism of the CPMV+CPA combination therapy, we characterized the immune cell population within the TME by flow cytometry. We evaluated innate immune cell infiltration 8 days after the first treatment. CPMV alone or in combination with CPA promoted the infiltration of the primary tumor by dendritic cells and macrophages, as indicated by the increased abundance of markers CD11c (**Figure** **5**A) and F4/80 (Figure S8, Supporting Information). This agrees with our previous study focusing on dermal melanoma immunotherapy with CPMV.^21^ The infiltration of innate immune cell was expected and reflects the immunogenic and adjuvant nature of the CPMV particle. Although CPMV is not infectious in mammals, it nevertheless alerts the immune system via toll‐like receptor signaling and therefore promotes the recruitment of innate immune cells. To investigate whether the infiltrating dendritic cells were activated, we measured the abundance of the co‐stimulatory molecule CD86, a marker of T cell priming. Accordingly, we found that CPMV increased the population of activated dendritic cells (CD11c/CD86^+^) in the primary tumor (Figure 5B). Although no statistical significance was observed, CPMV+CPA combination therapy trended to increase the population of activated dendritic cells more than CPMV alone or the CPMV+MFA combination therapy. Activated dendritic cells can present antigens and stimulate T cells. We therefore characterized the T cell population 13 days after the first treatment. We found that the CD4^+^ T cell population in the primary tumor was more abundant after CPMV monotherapy or combination therapy compared to the PBS group (Figure 5C), with CPMV monotherapy achieving a more significant increase. However, there was no significant difference among the various treatment groups in terms of the CD8^+^ T cell population in the primary tumor at this time point (Figure 5D). In the distant tumor, the CD4^+^ T cell population was significantly higher following CPMV monotherapy and CPMV+CPA combination therapy (Figure 5E), but the CD8^+^ T cell population was higher only following the CPMV+CPA combination therapy (Figure 5F). Together, these results indicated that CPMV+CPA combination therapy induced the recruitment of innate immune cells in the primary tumor, increased the activation of dendritic cells, and induced tumor‐infiltrating CD4^+^ and CD8^+^ T cells to establish adaptive immunity.

### 2.7 Immunohistochemistry

**Figure 6:**
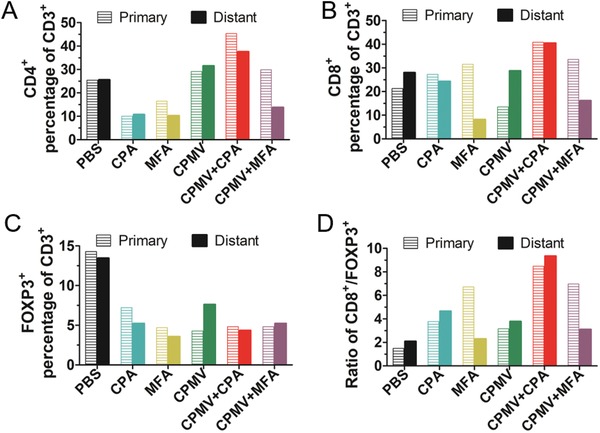
CPMV+CPA combination therapy systemically reversed the immunosuppression. Intratumoral A) CD4^+^, B) CD8^+^, and C) FOXP3^+^ cells in the percentage of CD3^+^ T cells. Number of positive cells was determined by immunochemical staining. D) CD8^+^/FOXP3^+^ cells ratio.

To gain deeper insight into the nature of the tumor‐infiltrating immune cells, tumors harvested from the 4T1 bilateral model on day 13 were fixed in 10% v/v phosphate‐buffered formalin and embedded in paraffin for immunohistochemical analysis. Sections were stained with anti‐CD3, anti‐CD4, anti‐CD8, and anti‐FOXP3 antibodies (Figure S9, Supporting Information). Substantial numbers of CD3^+^ T cells infiltrated into both the primary and distant tumors. We found that the primary tumor in the CPMV monotherapy group contained more CD3^+^ T cells than the PBS control. However, the distant tumor in the CPMV+CPA combination therapy showed less CD3^+^ T cells (Figure S9A, Supporting Information). The primary tumor treated with CPMV and its combinations contained more CD4^+^ T cells than the distant tumor, suggesting CPMV can directly recruit CD4^+^ T cells into the tumor microenvironment (Figure S9B, Supporting Information). Similar trends with flow cytometry analysis were observed when looking the CD4^+^ T cells in the percentage of CD3^+^ T cells. The CD4^+^ T cells in the percentage of CD3^+^ T cells were decreased by CPA or MFA chemotherapy, and increased by CPMV monotherapy or CPMV+CPA combination therapy, for both primary and distant tumors (**Figure** **6**A). The CD8^+^ T cells in the percentage of CD3^+^ T cells were increased by CPMV+CPA combination therapy for both primary and distant tumors (Figure 6B). The ratio of CD8^+^/CD4^+^ T cells increased by CPA monotherapy for both primary and distant tumors, and this was also observed by MFA monotherapy, but only for the primary tumor (Figure S10, Supporting Information). Interestingly, both CPMV+CPA and CPMV+MFA combination therapies did not change the ratio of CD8^+^/CD4^+^ T cells (Figure S10, Supporting Information). Both the primary and distant tumors in the PBS group contained large populations of FOXP3^+^ cells (Figure 6C), suggesting that 4T1 tumors are populated with immunosuppressive regulatory T (T_reg_) cells. The CPA, MFA, and CPMA monotherapies and the combination therapies depleted the T_reg_ cell populations in both the primary and distant tumors (Figure 6C). Given that tumor‐infiltrating T cells are the main antitumor response effectors, by the infiltration of CD4^+^ and CD8^+^ T cells and depletion of T_reg_ cells to reverse the immunosuppression may explain the synergistic efficacy of the CPMV+CPA combination therapy. To reflect the reverse of immunosuppression by CPMV+CPA combination therapy, we analyzed the CD8^+^/FOXP3^+^ cells ratio, which has been suggested to reflect the antitumor immunity more strongly than simply the numbers of CD8^+^ and FOXP3^+^ T cells.^35^, ^36^ As shown in Figure 6D, only CPMV+CPA combination therapy is capable of increasing the CD8^+^/FOXP3^+^ ratio in both primary and distant tumors, suggesting the immunosuppression was systemically reversed, which may explain the basis of their synergistic efficacy in the treatment of bilateral 4T1 tumors.

## 3 Discussion

New therapies or drug combinations that achieve an effective and durable treatment response are needed for treatment of metastatic TNBC, and antitumor immunity has been recognized as a promising strategy of such treatments. It may be necessary to kill tumor cells using an optimal chemotherapy regimen while also stimulating an antitumor immune response to eliminate residual tumor cells or keep them in check.^37^ In this study, we found that CPMV‐based in situ vaccination combined with systemic low‐dose CPA chemotherapy achieves impressive synergistic efficacy against 4T1 tumors. Although without complete regression, the combination therapy not only significantly inhibited the growth of primary tumors and substantially improved survival, it also showed similar efficacy against a distant tumor, and suppressed 4T1 lung metastasis, therefore showing an impressive abscopal effect. Mechanistic analysis indicated that the CPMV+CPA combination therapy activated APCs, systemically reversed the immunosuppression by inducing tumor‐infiltrating T cells and depleting the immunosuppressive T_reg_ cell. Abscopal effects with complete regression of the distant tumors has been observed in treatment of several tumor models by combination therapies,^38^ particularly when the immune checkpoint inhibitors or stimulators were included.^39^ However, in the treatment of TNBC (such as 4T1 model), complete regression is not typically observed,^39^ probably due to the aggressive nature of TNBC.

While oncolytic viruses have been explored for cancer immunotherapy,^40^ and T‐VEC has been approved for clinical use,^41^ we have turned toward plant virus–based nanotechnologies.^42^ Specifically, we chose CPMV for immunotherapy because i) CPMV as a plant virus is noninfectious to mammals and thus can be considered as safer compared to mammalian pathogens; ii) the production of CPMV through molecular farming enables highly scalable manufacture while keeping production costs at economic levels; iii) unlike the oncolytic viruses, CPMV can induce antitumor immune responses without any other cytotoxicity. In our preliminary experiments, CPMV in situ vaccination showed limited efficacy against 4T1 tumors regardless of the dose escalation. This modest efficacy was not as promising as previous results in murine melanoma and ovarian cancer,^19^, ^20^, ^21^ probably due to the weakly immunogenic and highly aggressive nature of the 4T1 tumors.^43^ Another potential explanation for the lower efficacy of CPMV against 4T1 tumors compared to B16F10 melanoma and ID8/Vegf/Defb29 ovarian tumors is the different mouse strains used as tumor models. The BALB/c strain has a Th2 bias whereas the C57BL6 strain has a Th1 bias, which may promote more effective T cell responses.^44^ To improve the efficacy, we developed a combination therapy strategy using CPMV and low‐dose CPA based on the concepts of a) CPMV as an in situ vaccine to efficiently reverse local tumor‐mediated immunosuppression in the TME and recruit antitumor immune effector cells, particularly innate immune cells, and b) CPA for the induction of ICD, increasing the immunogenicity of the 4T1 cancer cells. The pro‐immunogenic cancer cells induced by chemotherapy could then be targeted by the innate immune cells to trigger a systemic antitumor immune response. Our results indicated that CPMV+CPA combination therapy achieved the synergistic efficacy to attenuate the primary tumor growth and thus extended survival, with similar efficacy against a distant tumor, and also showed enhanced efficacy in reducing lung metastasis. Notably, the combination of CPMV and MFA did not show synergistic efficacy against the distant tumor, suggesting the antitumor immune response may be dependent on drug‐induced ICD. Cytokine profiling indicated that the secretion of IL‐12 in both the primary and distant tumors increased significantly in response to the combination therapy, suggesting a central role for IL‐12 that will be tested in future studies. CPA was previously shown to induce a Th2/Th1 shift in cytokine production, and accordingly we observed that IL‐12 together with the Th1 cytokine IFN‐γ was also secreted in greater amounts following treatment with CPA. However, the CPMV+CPA combination therapy may enhance this Th1 antitumor immune response, because much more IFN‐γ was secreted when CPMV and CPA were combined. We also found that the secretion of IFN‐α increased in both the primary and distant tumors in response to the combination therapy. CPA can synergize with type I interferons via systemic dendritic cell reactivation and the induction of immunogenic tumor apoptosis.^24^ Therefore, the enhanced secretion of IFN‐α could help to activate the dendritic cells, in turn inducing the secretion of IL‐12. The activation of dendritic cells was confirmed by flow cytometry, which revealed the expansion of a population of CD11c^+^CD86^+^ dendritic cells in response to the combination therapy. We also found that tumor‐infiltrating CD4^+^ and CD8^+^ T cells were more abundant after combination therapy, particularly in the distant tumor, which correlated with the presence of more IFN‐γ in the tumor microenvironment, but the ratio of CD8^+^/CD4^+^ T cells was not changed by the combination therapy. These results suggest the combination therapy rapidly induced a cytotoxic T cell response to promote tumor regression, particularly regression of the distant tumor. However, we observed an increased CD8^+^/CD4^+^ T cell ratio by the CPA monotherapy in both primary and distant tumors, but only in the primary tumor by the MFA monotherapy, implying the chemotherapy is toxic to the CD4^+^ T cells, including the T_regs_. Our data also indicated that the greater abundance of tumor‐infiltrating T cells in response to the CPMV+CPA combination therapy was associated with the depletion of the immunosuppressive T_reg_ cell population in the tumor microenvironment, and important aspect of many successful cancer immunotherapy approaches. Low doses of CPA appear to be highly toxic toward T_reg_ cells,^45^, ^46^ but our data indicated that CPMV can also induce T_reg_ depletion, which has not been reported before. Although the CPMV+CPA combination therapy did not completely eradicate the 4T1 tumors, our findings could nevertheless benefit breast cancer patients if similar efficacy is observed in clinical trials. Even though complete tumor regression has been observed by other combination strategies,^47^ one common observation is that TNBC is usually more aggressive and more challenging to cure.^39^ For better efficacy, triple therapy by adding a checkpoint inhibitor or stimulator to our combination therapy may help to amplify the antitumor immune response and achieve complete tumor regression. Particularly, given the positive results observed in clinical trials with anti‐PD1 therapy in metastatic breast cancer,^12^, ^13^ and the fact that CPA is part of the standard of treatment care, the therapeutic approach presented here may improve the efficacy of treatments for TNBC and other forms of cancer.

In conclusion, we have developed a CPMV+CPA combination therapy for the treatment of metastatic TNBC. The combination therapy not only achieved synergistic efficacy against the primary tumor that was directly vaccinated with CPMV, but also inhibited the growth of a distant tumor and the likelihood of lung metastasis. Our mechanistic analysis indicated that CPMV+CPA combination therapy activated APCs, systemically reversed the immunosuppression by inducing tumor‐infiltrating T cells and depleting the immunosuppressive T_reg_ population. The combination of CPMV and CPA thus has the potential to become a potent immunotherapeutic strategy with clinical benefits for TNBC patients.

## 4 Experimental Section

*Reagents*: CPA was purchased from Abcam and was dissolved in PBS. MFA was purchased from Santa Cruz Biotechnology and was dissolved in PBS immediately prior to use. RPMI‐1640 medium and Matrigel were purchased from Corning Life Sciences. Fetal bovine serum was purchased from Atlanta Biologicals, and penicillin/streptomycin was purchased from Thermo Fisher Scientific. All other chemical reagents were purchased from Sigma‐Aldrich.

*Virus, Cells, and Mice*: CPMV was produced by molecular farming,^48^ and purified following established procedures with modifications.^49^ Purified CPMV was suspended and stored in 0.1 m potassium phosphate buffer (pH 7.0) and was diluted in PBS where necessary.

Mouse 4T1 cells were purchased from ATCC (CRL‐2539) and were maintained in RPMI‐1640 medium supplemented with 10% v/v fetal bovine serum and 1% v/v penicillin/streptomycin. The cells were incubated at 37 °C in a 5% CO_2_ atmosphere.

BALB/c mice (female, 6 weeks old at the beginning of each experiment) were obtained from The Jackson Laboratory and were bred in‐house (School of Medicine, Case Western Reserve University, Cleveland, OH, USA). Animals were housed in groups with unlimited access to food and water. All mouse studies were performed in compliance with the Institutional Animal Care and Use Committee of Case Western Reserve University.

*Mouse Tumor Model and Treatment of Mice*: The 4T1 cells were harvested and resuspended in RPMI‐1640 medium at a concentration of 1 × 10^5^ cells mL^−1^ and mixed 1:1 v/v with Matrigel at 4 °C. Mice were injected s.c. with 100 µL of the mixture (including 5 × 10^4^ cells) into the right flank, or were injected in both flanks for the bilateral model. Tumors were allowed to grow for 8–10 days until the size reached 30–50 mm^3^ before randomization and treatments. CPA was administered i.p. with an injection volume of 200 µL, whereas MFA and CPMV were administered i.t. with an injection volume of 20 µL. For the CPMV+MFA combination therapy, the two agents were mixed and delivered together with an injection volume of 20 µL. Three treatments were administered at intervals of 7 days. Tumors were measured at least every other day using digital calipers. The tumor size (in cubic millimeters) was calculated using the formula: (width^2^ × length)/2. When the tumor size (either primary or distant tumor in the bilateral model) reached 1000 mm^3^, the mice were euthanized.

*Lung Metastasis Analysis*: Eight days after the first treatment, mice (*n* = 4) treated in the bilateral model were euthanized, and the lungs were collected and digested with collagenase type IV to form a single‐cell suspension. The cells were cultured with 60 × 10^−6^ m 6‐thiogunine for 10 days. The colonies formed by clonogenic metastatic 4T1 cancer cells were then fixed with methanol and stained with 0.1% crystal violet.^32^ For quantification, the crystal violet stained colonies were dissolved with 10% acetic acid and the absorbance at 590 nm was measured and normalized to the PBS control group. The single lung cells were stained with a cocktail of Pacific Blue antimouse CD45 (clone 30‐f11, BioLegend), FITC antimouse CD11b (clone M1/70, BioLegend), and PE/Cy7 antimouse Ly‐6G/Ly‐6C (Gr‐1) (clone RB6‐8C5, BioLegend), and then analyzed by flow cytometry. The total lung cell population was first gated on CD45, then CD11b and Gr‐1. On days 8 and 13 after the first treatment, mice (*n* = 2) were euthanized, the lungs were harvested and fixed with neutral‐buffered formalin solution (Sigma‐Aldrich), and then sectioned for H&E staining. Slides were scanned with ZEISS AxioScan Z1 slide scanner using 20×/0.8NA objective.

*Cytokine Assay*: The following ELISA kits were used to detect the cytokines based on the manufacturer's recommendations: IFN‐γ Mouse ELISA Kit (BMS606TWO), IFN‐α Mouse ELISA Kit (BMS6027TWO), and IL‐12 p70 Mouse Uncoated ELISA Kit with Plates (88‐7121‐22). Tumor‐bearing mice (*n* = 3) were treated with CPMV, CPA, MFA, and combinations as described above. Tumors were harvested on days 8 and 13 after the first treatment and pooled together as groups. Tumors were sliced, resuspended in HBSS buffer containing complete protease inhibitor (Roche) at a concentration of 2 mg mL^−1^, and then homogenized at 4 °C. The samples were centrifuged at 10 000 × *g* and the supernatants were used to measure IL‐12, IFN‐α, and IFN‐γ levels in quadruplicate using the ELISA kits listed above. The total protein content of the supernatant was determined using the Pierce BCA protein assay kit (Thermo Fisher Scientific) following the protocol provided by the manufacturer. The cytokine levels were normalized to the total protein content.

*Flow Cytometry*: The following antibodies and reagents were used for flow cytometry, all obtained from BioLegend: Pacific Blue antimouse CD45 (clone 30‐F11), FITC antimouse CD11b (clone M1/70), APC antimouse CD11c (clone N418), PE antimouse F4/80 (clone BM8), Brilliant Violet 605 antimouse CD86 (clone GL‐1), Alexa Fluro 700 antimouse I‐A/I‐E (clone M5/114.15.2), APC/Cy7 antimouse CD3 (clone 145‐2C11), FITC antimouse CD4 (clone Gk1.5), APC antimouse CD8 (clone 53‐6.7), Alexa Fluor 700 antimouse CD25 (clone PC61), PE antimouse FOXP3 (clone MF‐14), Zombie yellow fixable viability kit, and antimouse CD16/32 (clone 93). Tumor bearing mice (*n* = 3) were treated with CPMV, CPA, MFA, and combinations as described above. Tumors were harvested on days 8 and 13 after first treatment and pooled together as groups. Single‐cell suspensions were prepared as previously described^19^ and incubated for 15 min at 4 °C with a CD16/CD32 antibody (diluted in PBS) to block Fc receptors before washing with PBS. Tumor cells harvested on day 8 were tested using the innate panel and were incubated at 4 °C in triplicate with the cocktail of zombie yellow viability, CD45, CD11b, CD11c, F4/80, CD86, and I‐A/I‐E antibodies diluted in PBS. Tumor cells harvested on day 13 were tested using the adaptive panel and were incubated at 4 °C in triplicate with the cocktail of zombie yellow viability, CD45, CD3, CD25, CD4, CD8, and FOXP3 antibodies. Cells were washed twice with PBS and then fixed with 3% v/v paraformaldehyde for flow cytometry using an LSR II (BD Biosciences). The data were analyzed using the FlowJo v8.6.3 software.

*Immunohistochemistry*: The following antibodies were used for immunohistochemistry: Ki‐67 antibody (clone SP6, Invitrogen), anti‐CD3 antibody (clone SP7, Abcam), anti‐CD4 antibody (clone 4SM95, eBioscience), anti‐CD8 antibody (clone 4SM15, eBioscience), and anti‐FOXP3 antibody (clone FJK‐16s, eBioscience). For immunohistochemistry, lungs were harvested on day 8 and tumors were harvested on day 13 after the first treatment, fixed in 4% v/v paraformaldehyde in PBS for 2 days, and then transferred to 1% v/v paraformaldehyde in PBS. Sectioning and staining were carried out by the Case Western Reserve University Pathology Core and Moores Cancer Center Histology Core (University of California, San Diego). Fixed tumors were equilibrated serially in 10% v/v neutral‐buffered formalin (twice), then 70% and 80% v/v ethanol at 45 °C for 90 min each, then in 95% and 100% v/v ethanol at 45 °C for 2 h each (three times), then in xylene at 45 °C for 90 min (twice), and finally in paraffin for 2 h at 45 °C (three times). The samples were stored at 60 °C prior to embedding in paraffin. Paraffin blocks were soaked in cold distilled water followed by the preparation of 5 × 10^−6^ m sections using a microtome. Sections were placed on a charged slide at 45 °C in distilled water, and were allowed to dry overnight at room temperature. The slides were baked at 60 °C for 75 min and then deparaffinized in xylene twice for 7 min before rehydration through 100% ethanol (twice), 95% ethanol (twice), and 70% ethanol for 2 min followed by a rinsing in distilled water. Antigen retrieval was achieved using 0.01 m citrate buffer at pH 6.0 and then the slides were placed in a pressure cooker for 30 s at 123 °C. After cooling down, the slides were rinsed in distilled water for 2 min. The endogenous peroxidase activity was blocked in Peroxidazed (PX968M, BioCare Medical) for 8 min, followed by rinsing in distilled water. The endogenous mouse IgG was blocked using Rodent Block (RBM961, BioCare Medical) for 20 min, followed by rinsing with Tris‐buffered saline containing Tween‐20 (TBST). The slides were then stained with the antibodies listed above or without antibodies as negative controls for 1 h at room temperature, followed by rinsing in TBST. The slides were then treated with Rabbit‐on‐Rodent HRP Polymer (RMR622H, BioCare Medical) for 30 min and rinsed with TBST. The slides were incubated with the Betazoid DABKit (BioCare Medical) for 5 min in the dark. After rinsing with distilled water, the slides were counterstained with CAT hematoxylin (CATHE‐M, BioCare Medical). Slides were scanned with ZEISS AxioScan Z1 using 20×/0.8NA objective. Image analysis was done in QuPath^50^ using positive cell detection for Ki‐67, FOXP3, CD3, CD4, and random forest classifier for CD8. Cells positive for immunolabeled proteins were identified based on threshold above background and characteristic staining pattern (nuclear in Foxp3 and Ki67; membrane in CD3, CD4, and CD8). Areas of necrotic cores were excluded from the analysis. The number of positive cells per square millimeter was automatically calculated in QuPath software. Representative regions were selected to match the average density of positive cells in the sample.

*Statistical Analysis*: Data were analyzed and charts were generated using Prism 5 (GraphPad Software). Statistical significance was determined by two‐way or one‐way analysis of variance (ANOVA). Survival data were compared with the Mantel–Cox (log‐rank) and/or Gehan–Breslow–Wilcoxon test. In the figures, asterisks represent the following *p*‐values: *p < 0.05, **p < 0.01, and ***p < 0.001.

## Conflict of Interest

The authors declare no conflict of interest.

## Supporting information

Supplementary
