# Supplementary material for: Cowpea Mosaic Virus Immunotherapy Combined with Cyclophosphamide Reduces Breast Cancer Tumor Burden and Inhibits Lung Metastasis
Source: Adv Sci (Weinh). 2019 Jun 19;6(16):1802281. doi: 10.1002/advs.201802281 (PMC6702650; doi:10.1002/advs.201802281)
Supplement: Supplementary file 1 — Supplementary [file ADVS-6-1802281-s001.pdf]

Copyright WILEY-VCH Verlag GmbH & Co. KGaA, 69469 Weinheim, Germany, 2019.

ADVANCED SCIENCE
Open Access

Supporting Information

for *Adv. Sci.,* DOI: 10.1002/advs.201802281

Cowpea Mosaic Virus Immunotherapy Combined with Cyclophosphamide Reduces Breast Cancer Tumor Burden and Inhibits Lung Metastasis

*Hui Cai, Chao Wang, Sourabh Shukla, and Nicole F. Steinmetz**

Copyright WILEY-VCH Verlag GmbH & Co. KGaA, 69469 Weinheim, Germany, 2019.

Supporting Information

Cowpea mosaic virus immunotherapy combined with cyclophosphamide reduces breast cancer tumor burden and inhibits lung metastasis

*Hui Cai, Chao Wang, Sourabh Shukla, Nicole F. Steinmetz**

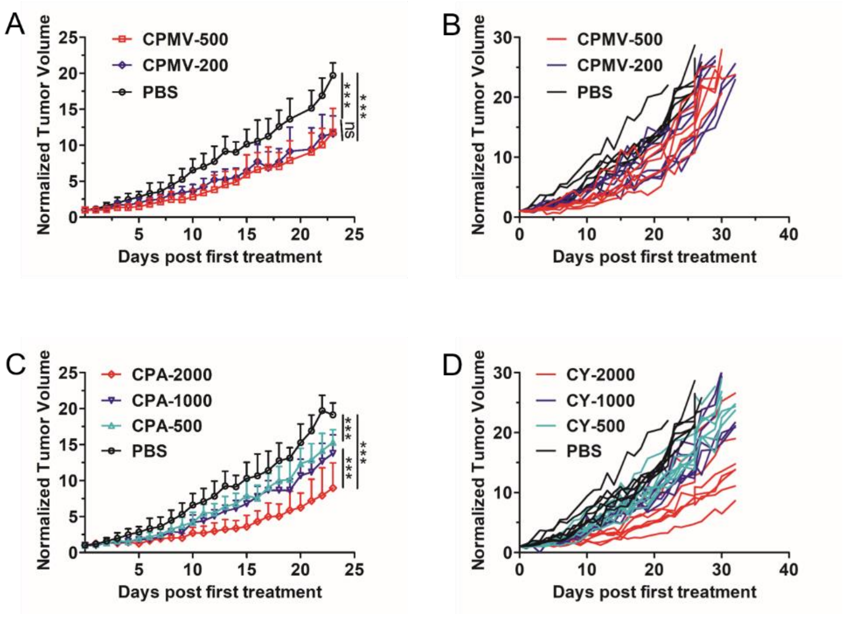

Figure S1. CPMV or CPA monotherapy for the treatment of 4T1 tumors. A) Tumor growth curves following CPMV (i.t.) doses of 200 and 500 µg. B) Individual tumor growth kinetics following CPMV treatment. C) Tumor growth curves following CPA (i.p.) doses of 500, 1000 and 2000 µg. D) Individual tumor growth kinetics of CPA treatment. For tumor growth curves, the average normalized tumor volume and stand deviations are shown, with statistical analysis by two-way ANOVA (*** $p < 0.001$, ns = not significant).

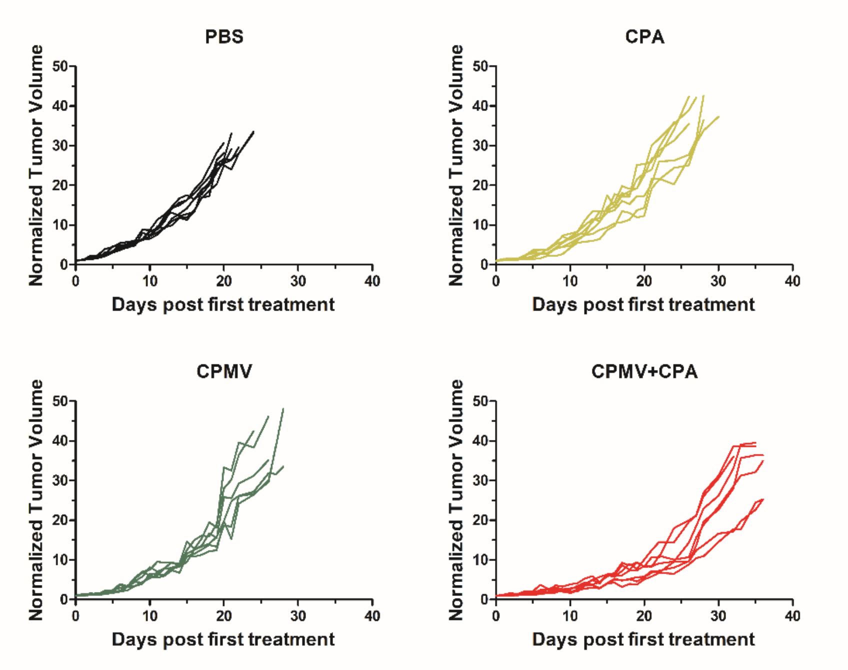

Figure S2. Individual tumor growth kinetics following treatment with CPMV (200 µg), CPA (500 µg) and a combination of CPMV (200 µg) + CPA (500 µg), corresponding to the average tumor growth curves of in Fig. 1D.

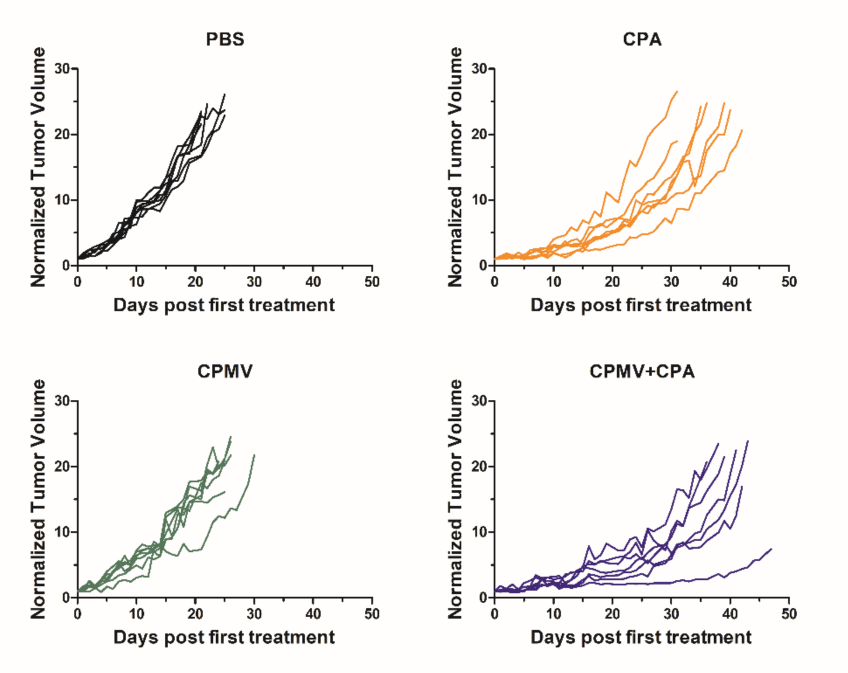

Figure S3. Individual tumor growth kinetics following treatment with CPMV (200 µg), CPA (2000 µg) and a combination of CPMV (200 µg) + CPA (2000 µg), corresponding to the average tumor growth curves of in Fig. 1E.

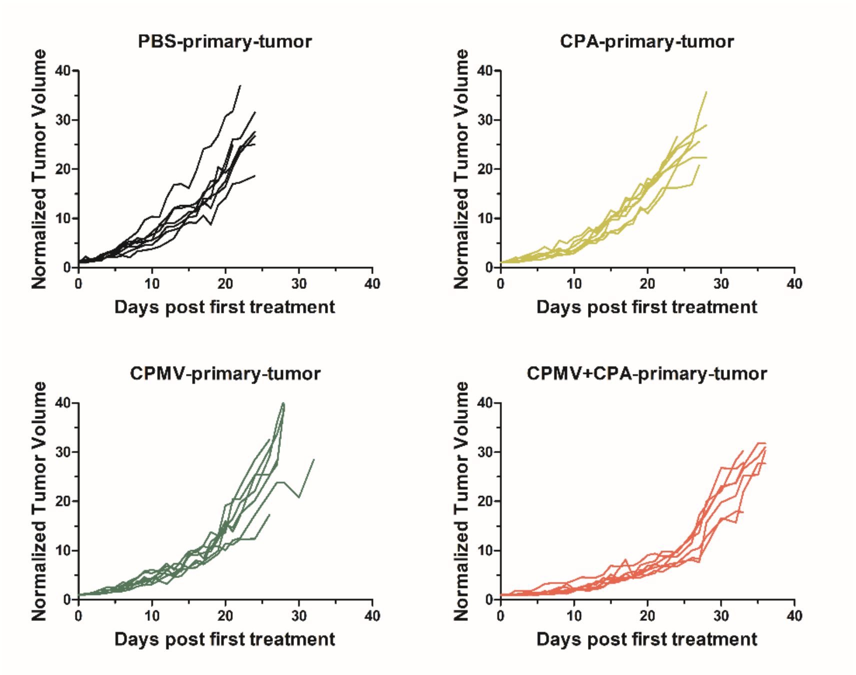

Figure S4. Individual primary tumor growth kinetics in the bilateral 4T1 model following treatment with CPMV and CPA, corresponding to the average tumor growth curves in Fig. 1F (primary tumor).

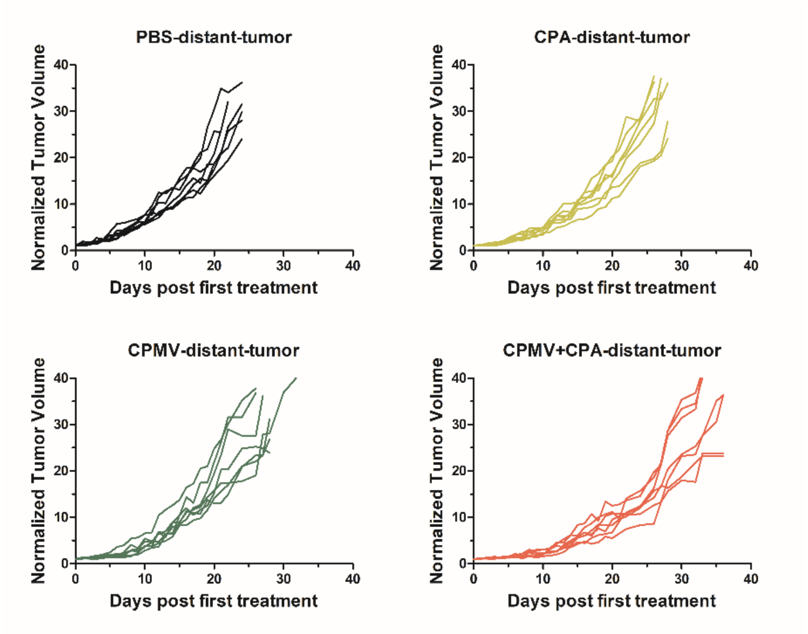

Figure S5. Individual distant tumor growth kinetics in the bilateral 4T1 model following treatment with CPMV and CPA, corresponding to the average tumor growth curves of in Fig. 1F (distant tumor).

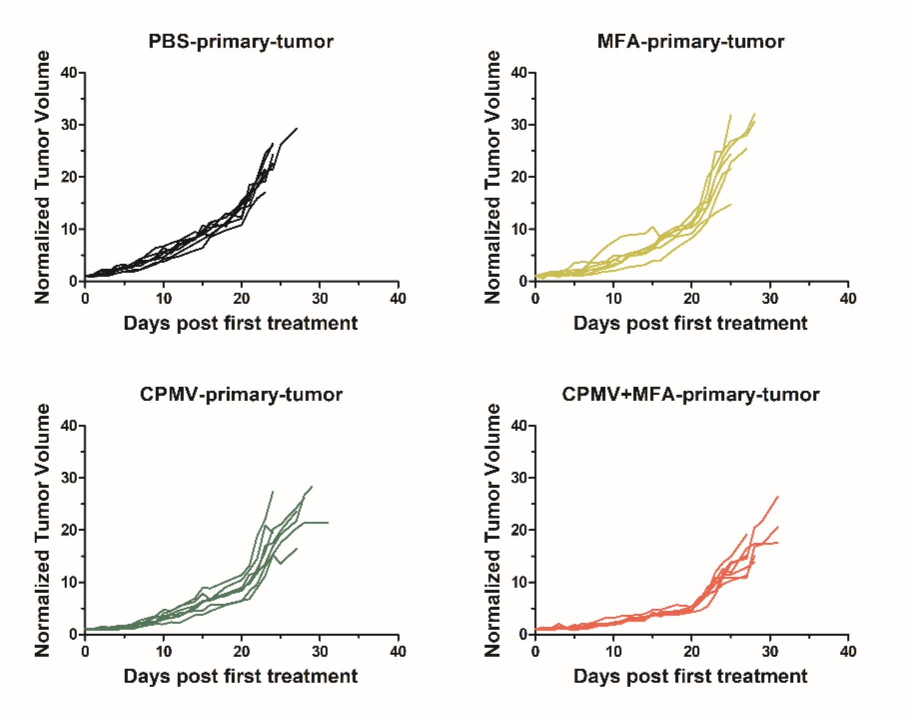

Figure S6. Individual primary tumor growth kinetics in the bilateral 4T1 model following treatment with CPMV and MFA, corresponding to the average tumor growth curves of in Fig. 2C (primary tumor).

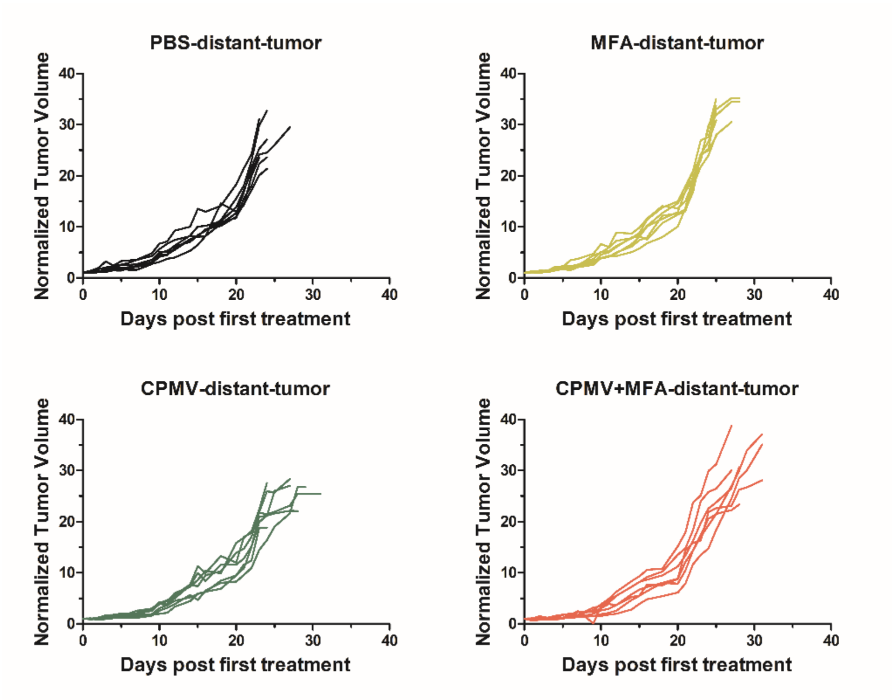

Figure S7. Individual distant tumor growth kinetics in the bilateral 4T1 model following treatment with CPMV and MFA, corresponding to the average tumor growth curves of in Fig. 2C (distant tumor).

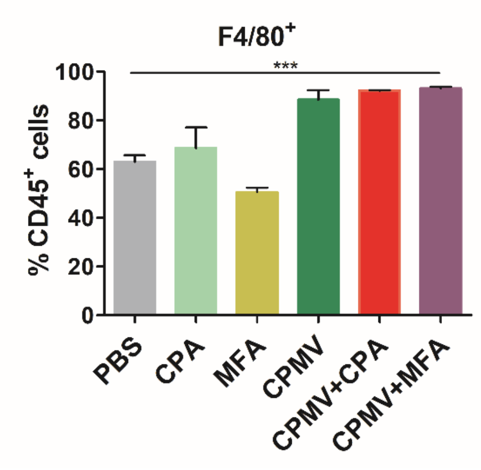

Figure S8. Tumor-infiltrating macrophages on day 8 determined by flow cytometry, with statistical analysis by one-way ANOVA and Tukey's test (*** p< 0.001).

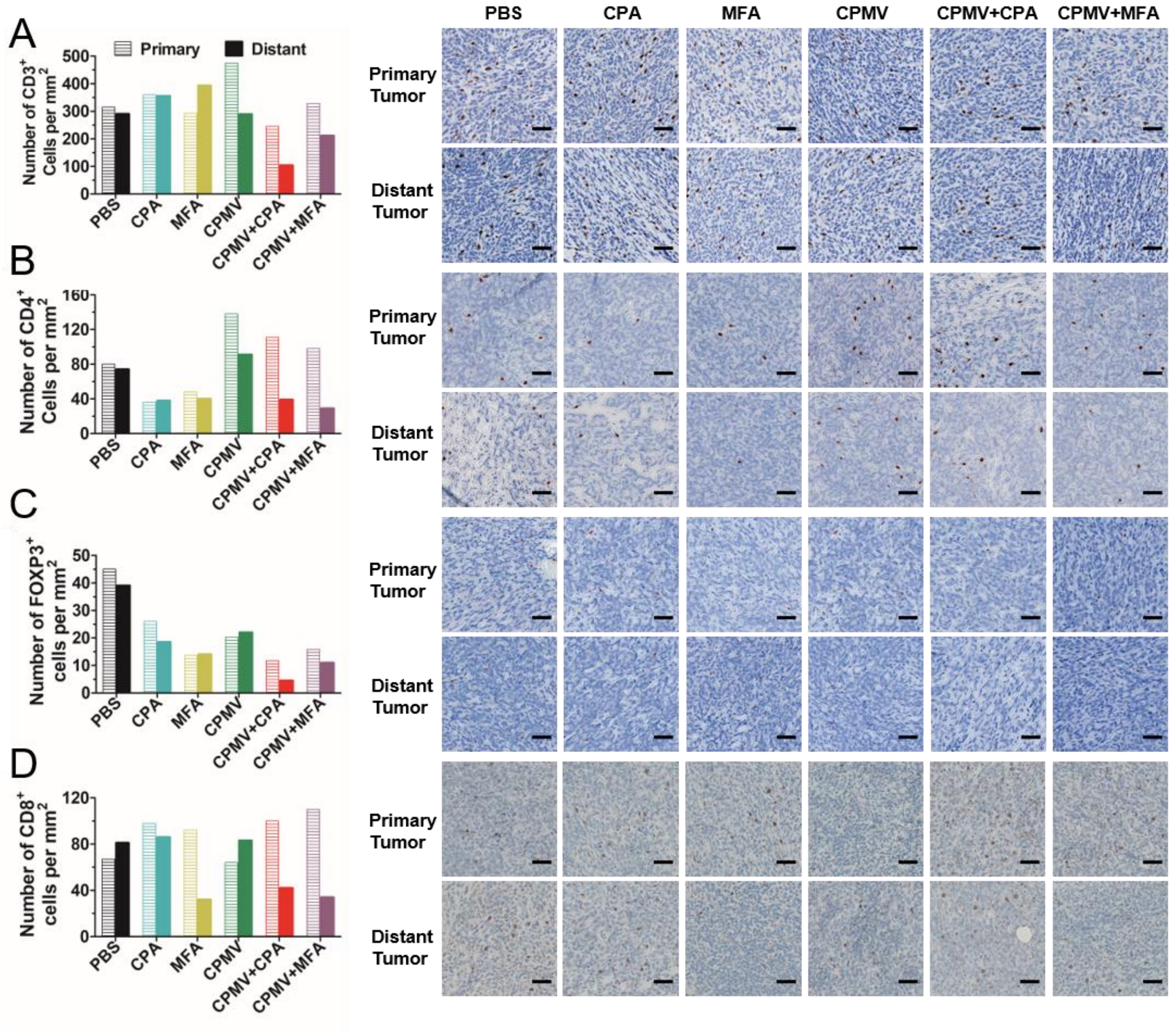

Figure S9. Immunohistochemical staining of tumors 13 days after the first treatment. CD3 (a), CD4 (b), CD8 (c) and FOXP3 (d) were detected on paraffin-embedded tumor sections. Left: number of positive cells per mm^2. Right: representative tumor sections (Scale bar = 50 µm).

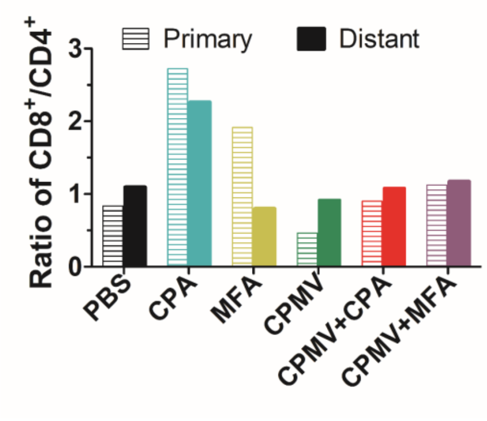

Figure S10. $CD8^+/CD4^+$ cells ratio (based on data from Figure S9).
